# Substrate Integrated Waveguide Cross-Coupling Filter with Multilayer Hexagonal Cavity

**DOI:** 10.1155/2013/682707

**Published:** 2013-12-25

**Authors:** B. Wu, Z. Q. Xu, J. X. Liao

**Affiliations:** Research Institute of Electronic Science and Technology, University of Electronic Science and Technology of China, Chengdu 611731, China

## Abstract

Hexagonal cavities and their applications to multilayer substrate integrated waveguide (SIW) filters are presented. The hexagonal SIW cavity which can combine flexibility of rectangular one and performance of circular one is convenient for bandpass filter's design. Three types of experimental configuration with the same central frequency of 10 GHz and bandwidth of 6%, including three-order and four-order cross-coupling topologies, are constructed and fabricated based on low temperature cofired ceramic (LTCC) technology. Both theoretical and experimental results are presented.

## 1. Introduction

Waveguide filters are widely used in various microwave and millimeter-wave communication systems, especially airborne platforms, communication satellites, earth stations, and wireless base stations, due to their high quality factor (*Q*-factor) and high power capability. However, they are bulky and not suitable for high-density integration, which greatly increases the cost of the entire system [1, 2]. Recently substrate integrated waveguide (SIW) which is synthesized in a planar substrate with arrays of metallic via provides a low-profile, low-weight, and low-cost solution while maintaining high performance [3, 4].

Recently, various types of SIW filters have been paid much attention. It is known that conventional structures of SIW filters are predominantly based on rectangular and circular cavities [5, 6]. Rectangular cavity is superior for its flexible structure while circular cavity is for its high performance [7–9]. To combine their predominance together, novel SIW filters with hexagonal resonators are proposed in [10]. Since any of the six sides of a hexagonal cavity can be utilized for coupling, it is flexible in the design of SIW filter. However, their physical sizes are not compact enough due to their planar and single-layer structure. Besides, it is usually not easy to implement negative coupling in planar structure owing to similar coupling manner between the adjacent resonators in the same plane.

In this paper, in regard to compact size and stringent frequency selectivity, multilayer hexagonal cavities which combine flexibility of rectangular cavities and performance of circular cavities and their applications to SIW filters are proposed. Three types of experimental filter configuration at the same central frequency of 10 GHz are fabricated using low temperature cofired ceramic (LTCC) technology. Design details are described, and both simulated and experimental results are presented and discussed.

## 2. Filter Analysis and Design

### 2.1. Hexagonal Resonant Cavity

**Figure 1:**
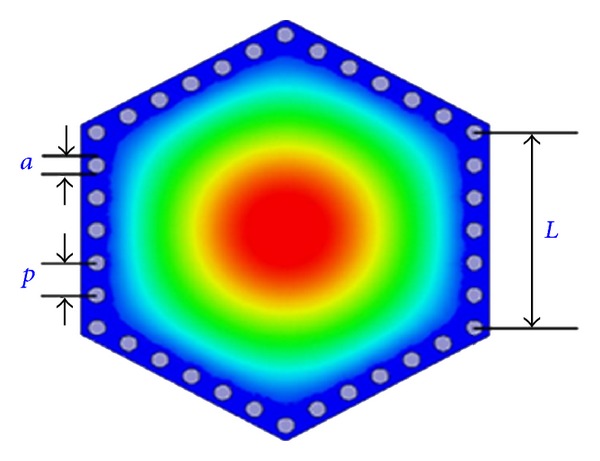
The configurations and electric field distributions of the fundamental mode of a hexagonal cavity resonator.

The configurations and electric field distributions of the fundamental mode of a hexagonal cavity resonator are shown in Figure 1. Its electric field distributions and resonant characteristics are similar with circular resonators.

Here, *L*, *a*, and *p* stand for length of regular hexagonal cavity, diameter of metallized via holes, and pitch between them, respectively. There is no accurate formula between geometrical parameters and eigenfrequencies of a hexagonal resonator yet, but [10] proposed an approximate expression as

(1) $$\begin{array}{c} L=\frac{c}{\sqrt{\varepsilon_{r}}}\cdot \frac{\mu^{\prime }}{2\pi f_{r}}. \end{array}$$

Here, *c*, *ε*_*r*_, and *f*_*r*_ stand for speed of light, relative permittivity of dielectric substrate and eigen frequencies of SIW hexagonal resonator, respectively. *μ*′ = 2.75 is the modified root coefficient based on Bessel function.

### 2.2. Analysis of the Proposed Filters

Taking three-order and four-order filters as examples, three types of novel multilayer SIW hexagonal cavity filters are proposed, with design goals illustrated in Table 1.

Abstracted coupling coefficients determine coupling patterns and initial geometrical dimensions, while calculating normalized frequency of prototype lowpass filter in advance as

(2) $$\begin{array}{c} \Omega =\frac{\omega_{0}}{\Delta \omega }\left(\frac{\omega }{\omega_{0}}-\frac{\omega_{0}}{\omega }\right). \end{array}$$

For Filters A and C, their coupling matrixes have negative elements on secondary diagonal, unlike direct coupling. Magnetic coupling pattern contributes to achieve cross-coupling in multilayer structure. For Filter A, S_11_ remains and S_21_ inverses while inversing M_13_, M_31_, M_12_, and M_21_ simultaneously. For Filter C, S_11_ remains and S_21_ inverses while inversing M_41_, M_12_, M_21_, and M_21_ simultaneously. Transformed coupling matrixes and their *Q*-factors are shown as follows:

(3) $$\begin{array}{c} M_{\text{A}}=\left[\begin{array}{ccc} 0.0082 & 0.0546 & 0.0341\\ 0.0546 & -0.0325 & 0.0546\\ 0.0341 & 0.0546 & 0.0082 \end{array}\right],\;\;{\text{Q}}_{\text{A}}=15.3857,\\ M_{\text{B}}=\left[\begin{array}{ccc} -0.0082 & 0.0546 & 0.0341\\ 0.0546 & 0.0325 & -0.0546\\ 0.0341 & -0.0546 & -0.0082 \end{array}\right],\;\;{\text{Q}}_{\text{B}}=15.3857,\\ M_{\text{C}}=\left[\begin{array}{cccc} 0 & -0.0435 & 0 & 0.0085\\ -0.0435 & 0 & 0.0384 & 0\\ 0 & 0.0384 & 0 & 0.0435\\ 0.0085 & 0 & 0.0435 & 0 \end{array}\right],\\ {\text{Q}}_{\text{C}}=19.5396. \end{array}$$

Normally, coupling coefficient is positively correlated with size of coupling window, which can be preliminarily determined by their relational curve graph.

**Figure 2:**
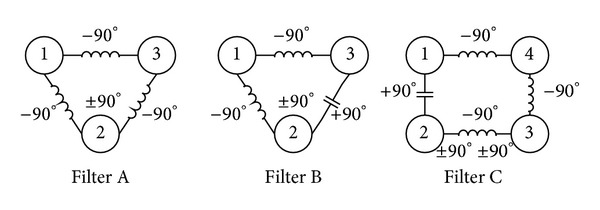
Coupling topological structures of Filters A, B, and C.

We define negative coupling coefficient as electric coupling and positive coupling coefficient as magnetic coupling. Numbered circle stands for each resonator, and the symbols of inductance and capacitance represent the opposite coupling relationships between two connective resonators, respectively. Hence, coupling topological structures of Filters A, B and C are shown in Figure 2.

For Filter A, signal passed primary channel obtains opposite phase against that passed cross-coupling channel and forms a transmission zero beyond passband. Similarly, Filter B forms a transmission zero underneath passband, while Filter C forms two at both outsides of pass band.

Magnetic coupling is achieved by slotting at the area with maximum magnetic field intensity on common surface between cavities, while electric coupling with maximum electric field intensity.

### 2.3. Parameters of the Proposed Filters

External *Q*-factors determined by input and output structures play a crucial role in suppressing return loss within pass band. Input and output adopt electric current probe form, which consist of GCPW and 50 Ω microstrip line. Width of GCPW's gap is assigned as 0.25 mm for preventing power leakage and machining tolerance.

**Figure 3:**
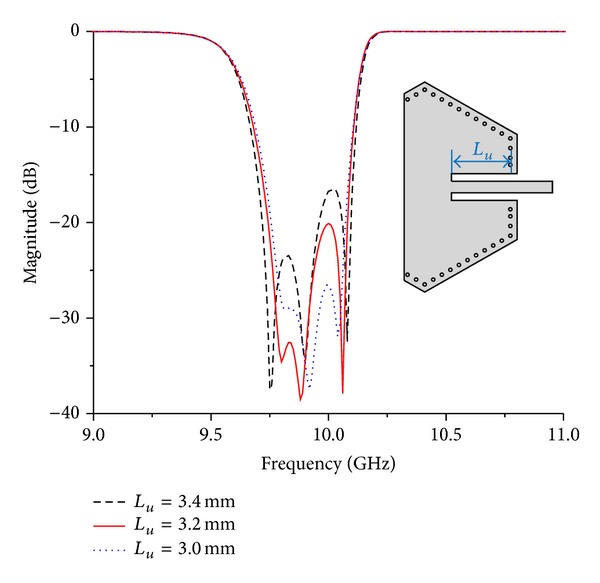
Effect of GCPW's length on return loss in Filter A.

Effect of GCPW's length on return loss in Filter A is shown in Figure 3. Loaded *Q*-factor varies due to length's change, causing variation of return loss within pass band. Hence, it is important for ports' dimensions to return loss within pass band.

**Figure 4:**
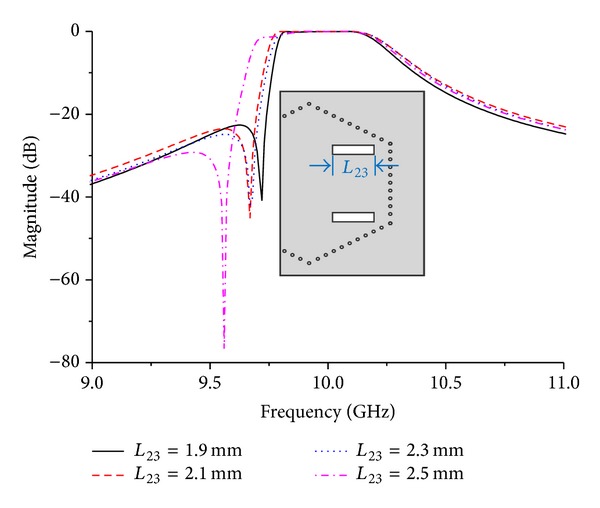
Effect of coupling window's length on transmission zero in filter B.

Transmission zero comes from cross-coupling when signal passed primary channel obtains 180° phase shift against that passed cross-coupling channel at corresponding frequency point. Therefore, cross-coupling has a great impact on transmission zero. Effect of coupling window's length on transmission zero in Filter B is shown in Figure 4. Coupling intensity between two resonant cavities is enhanced as length elongating, causing transmission zero underneath pass band to move downward.

**Figure 5:**
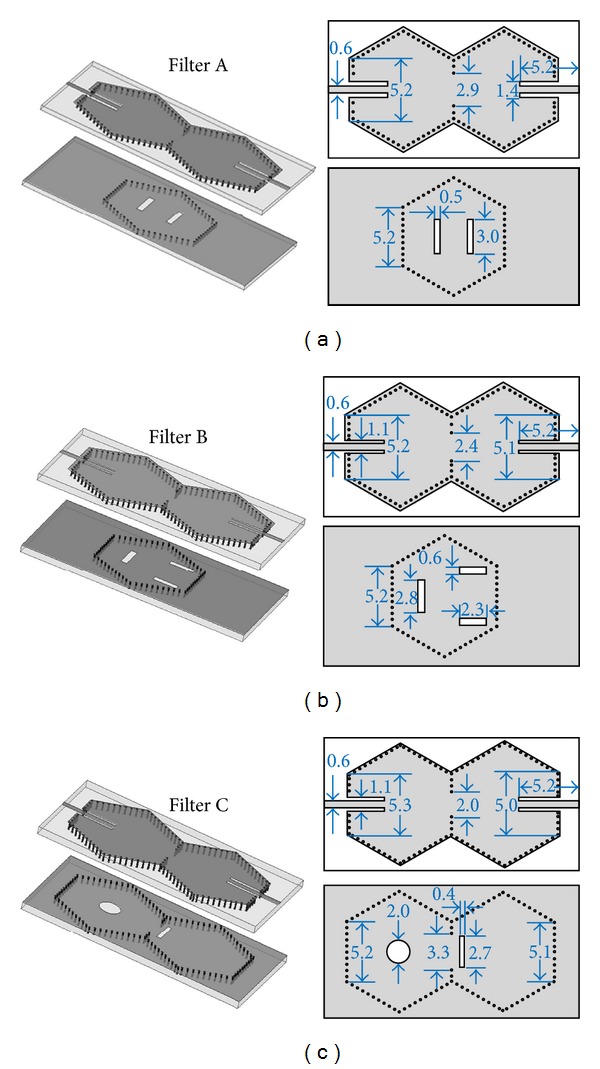
Final dimensions of Filters A, B and C. (unit mm).

**Figure 6:**
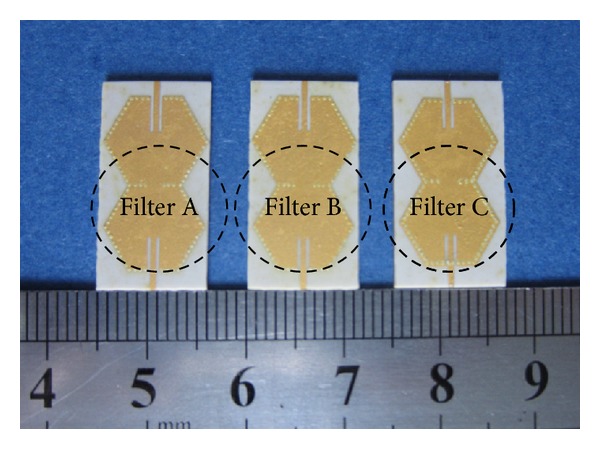
Photographs of fabricated Filters A, B and C.

After theory analysis, simulation and optimization, final dimensions and photographs of Filters A, B, and C are shown in Figures 5 and 6, respectively.

## 3. Fabrication and Experimental Results

The proposed three types of filters are implemented on a LTCC substrate with relative permittivity of 5.9, loss tangent of 0.0015, and thickness of 0.8 mm. Each resonant cavity, up and down surfaces of which being covered with gold, consists of four substrate layers within linear arrays of metallization via holes having diameter of 0.17 mm and center-to-center pitch of 0.45 mm.  Figure 6 shows the photograph of fabricated Filters A, B, and C. The external shapes of fabricated filters are 22 mm × 11.5 mm × 0.8 mm, 22 mm × 11.5 mm × 0.8 mm, and 22 mm × 12 mm × 0.8 mm, respectively.

**Figure 7:**
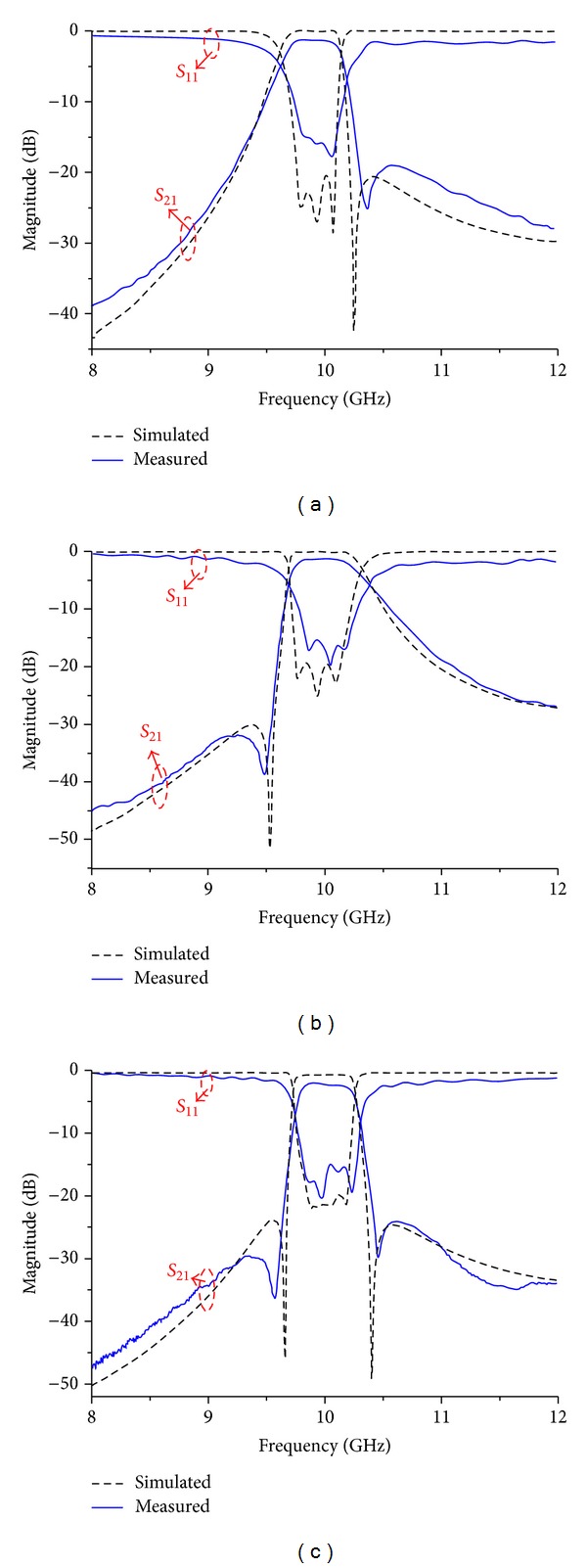
Simulated and measured frequency responses: (a) Filter A, (b) Filter B, and (c) Filter C, respectively.

An Agilent E8363B vector network analyzer is used for measurement. The simulated results and measured frequency responses are illustrated in Figure 7.

As can be seen, the measured results are in good agreement with the simulated ones. The measured responses of Filters A, B, and C have the fractional bandwidths of 5.83%, 5.95%, and 5.76%, respectively. The minimum passband insertion loss of Filter A is 1.82 dB, 1.68 dB for Filter B, and that of Filter C is 1.89 dB. The measured in-band return loss is greater than 16 dB for Filter A, 17 dB for Filter B, and 16 dB for Filter C, respectively.

By comparing the measured and simulated results, a small frequency shift and a little discrepancy in the in-band insertion loss are observed. The frequency shift is mainly caused by the difference between the actual and nominal values of the dielectric constant. The degeneration of the in-band insertion loss contributed to the test fixture and the manufacturing tolerance in LTCC process, including dielectric and printed conductor loss, wrinkles of conductor surface and edge, and cofireability of conductor and ceramic material. It should be noted that cofireability between conductor and ceramic during the manufacturing process is the main cause of the degeneration of the in-band insertion loss. If the material or process conditions of the ceramic and conductor are inappropriate, various macro and micro flaws may occur, which will worsen the in-band insertion loss. Meanwhile, in the cofiring process, minute pores may be found forming at the interface, which is yet another possible factor influencing the in-band insertion loss. To avoid the above undesirable possibilities, it is not sufficient only to optimize the cofiring process condition or profile. Rather, it is also necessary in some cases to revise the base powder of each material in order to improve the cofire ability behavior of the conductor and ceramic.

Overall, the proposed filter exhibits good frequency selectivity and compact size by profit from its modified ED and multilayer structure.  Table 2 shows the comparison with some other recent works on SIW filters. As can be seen, the proposed filter not only exhibits good selectivity owing to its flexible hexagonal cavities but also has a compact size by profit from multilayer LTCC structure.

## 4. Conclusion

Three types of novel SIW cross-coupling filters with multilayer hexagonal cavity based on LTCC technology are proposed and fabricated. N-2 transmission zeros in proposed n-order filers are achieved by introducing cross-coupling in multilayer substrate. The proposed filters have advantages of low insertion loss and good suppression at outband due to the flexible construction and relative high *Q*-factor of hexagonal resonant cavity, which demonstrate that the proposed filters have potential applications for high-density microwave communication.

## Figures and Tables

**Table 1:** Design goal of filters A, B, and C.

Filter types	Center frequency	Fractional bandwidth	Return loss	Transmission zeros
Filter A	10 GHz	6%	20 dB	10.3 GHz
Filter B	10 GHz	6%	20 dB	9.6 GHz
Filter C	10 GHz	6%	20 dB	9.6, 10.5 GHz

**Table 2:** Comparisons with some conventional SIW filters.

References	*f*_center_	BW	Occupying sizes
[10]	10 GHz	6%	>30 × 30 mm^2^
[11]	10 GHz	3.3%	>30 × 30 mm^2^
[12]	6 GHz	5%	>48 × 24 mm^2^
[13]	10 GHz	3.4%	>32 × 32 mm^2^
This work	10 GHz	6%	22 × 12 mm^2^
